# Reprogramming CAR T cells across genetic, epigenetic, metabolic and microenvironmental axes to improve efficacy and safety in cancer and autoimmune disease

**DOI:** 10.3389/fimmu.2026.1806459

**Published:** 2026-04-07

**Authors:** Marc Scherlinger

**Affiliations:** 1 Rheumatology Department, National Reference Center for Systemic Autoimmune Diseases (CNR RESO), Strasbourg University Hospital, Strasbourg, France; 2 UMR Inserm 1109 Immuno-Rhumatologie Moléculaire, Centre de recherche en biomédecine de Strasbourg (CRBS), Strasbourg, France

**Keywords:** autoimmunity, CAR T cells, CAR-Treg, exhaustion, genome editing, immunometabolism, safety switches, tumor microenvironment

## Abstract

CAR T-cell therapy has delivered durable remissions in several hematologic cancers, yet activity in solid tumors and extension to immune-mediated diseases remain constrained by recurring failure modes: imperfect antigen specificity, inadequate trafficking, progressive dysfunction under chronic stimulation, and toxic inflammatory syndromes. Early reports of CAR-based immune “resets” in refractory autoimmune disease amplify both promise and stakes, because acceptable risk is lower than in cancer and “on-target” effects may still be clinically unacceptable if they create long-term immunodeficiency. This review treats CAR T optimization as multi-layer reprogramming across genetic circuitry, epigenetic state, metabolism, and the tissue microenvironment. We argue that many celebrated single-layer upgrades (stronger signaling, checkpoint deletion, constitutive cytokine armoring) often trade one failure mode for another. Instead, the most credible path to simultaneously improving efficacy and safety is disciplined, failure-mode–driven design: (i) programmable antigen logic and titratable activation to reduce off-tissue damage; (ii) epigenetic programming that preserves renewable functional states without removing essential restraints; (iii) metabolic rewiring evaluated under physiologic stress conditions; and (iv) microenvironment-aware strategies that prioritize access and local control over brute-force potency.

## 1 Introduction

Chimeric antigen receptor (CAR-) T cells are living therapeutics whose behavior reflects the integration of receptor signaling, differentiation state, and environmental stress. Their clinical impact in B-cell malignancies established that redirected T cells can eradicate large disease burdens, but the same platform has struggled in most solid tumors where antigen heterogeneity, physical exclusion, suppressive cytokines, and nutrient deprivation combine to blunt function (1–3). Toxicities remain a second, independent ceiling: cytokine release syndrome (CRS) and immune effector cell–associated neurotoxicity syndrome (ICANS) emerge from inflammatory amplification loops that can be unpredictable and occasionally fatal, limiting dosing and narrowing target choice (4, 5).

Autoimmune diseases represent another disease group with huge unmet needs (6–8), but which change both the target definition and the risk calculus. In early clinical experiences, B-cell–directed CAR T cells have produced deep, drug-free remissions in severe, refractory systemic autoimmunity, consistent with an “immune reset” model in which autoreactive B-cell compartments are erased and immune reconstitution proceeds from naïve precursors (9–11). Antigen-specific approaches such as chimeric autoantigen receptor (CAAR) T cells aim for surgical deletion of pathogenic B cells while sparing the rest of the immune repertoire (12, 13). Alternatively, targeting auto-reactive B cell through 9G4 idiotype targeting using CAR-T cell appears a promising strategy (14). Finally, CAR-engineered regulatory T cells (CAR-Tregs) aim to restore tolerance rather than kill, but their therapeutic value depends on phenotypic stability and controlled suppression rather than maximal activation (15).

In both onco-hematology and autoimmunity, CAR-T therapy is approaching a ceiling set by incomplete eradication of the pathogenic compartment. In B-cell malignancies, CD19 CAR-T cells can induce profound B-cell aplasia and deep remissions, yet measurable/minimal residual disease persists in a subset of patients and relapse remains a recurring outcome (3). In systemic autoimmunity, early “immune reset” responses are striking, but the persistence of autoreactive B-cell clones in some cases—often reflected by sustained autoantibody production—offers a plausible route to relapse (9, 16). These observations point to a shared problem: current CAR-T products are frequently potent enough to debulk disease, but not consistently programmed to eliminate (or permanently control) the most resilient residual populations. Closing this gap—without simply escalating toxicity—will be essential to deliver durable remission across patients and may also accelerate progress in solid tumors, where hostile microenvironments and antigen constraints punish brute-force potency.

Compared with oncology, autoimmune indications impose a different therapeutic objective and a narrower tolerance for collateral damage. Early CD19-directed CAR-T studies in severe systemic autoimmune diseases have shown feasibility and substantial clinical responses, but the available datasets remain small, uncontrolled, and centered on B-cell-driven systemic disease (9, 13). At the same time, the field is already revealing disease-specific complexities: treatment-refractory autoimmune neuropathies have now been reported as candidate indications, relapse after CD19 CAR-T can still occur through persistent plasma-cell biology as illustrated by BCMA CAR-T rescue in inflammatory myositis (16), and organ-localized inflammatory toxicities such as local immune effector cell-associated toxicity syndrome (LICATS) suggest that efficacy and toxicity may co-localize within previously inflamed tissues (17). Clinical development is also diversifying beyond first-generation autologous anti-CD19 programs, with dual BCMA/CD19 and universal allogeneic anti-CD19/BCMA approaches entering clinical testing (18), alongside antigen-specific strategies. These observations argue that autoimmunity should not be treated as a simple extension of cancer CAR-T, but as a distinct design problem in which deep B cell depletion must be tailored to achieve autoreactive B cell removal while minimizing infection risk by achieving rapid B cell repopulation with a non-autoreactive repertoire (11).

In this review, we map the main engineering levers used to enhance CAR-T durability and clinical performance—CAR design and signaling logic, metabolic programming, and cellular fitness. We organize these approaches across genetic, epigenetic, metabolic, and microenvironment-informed interventions, and for each we ask two practical questions: what failure mode is being targeted (e.g., exhaustion, poor persistence, antigen escape, suppressive niches), and what trade-off is introduced (e.g., heightened inflammation, impaired control, or new escape routes). The main points are summarized in **Table 1**. This framework is intended to clarify which strategies plausibly improve both efficacy and safety, versus those that mainly redistribute risk.

**Table 1:** Possible modifications to counteract cause of failures of CAR-T cell therapies in cancer and autoimmunity.

Failure mode	Axis	Improvement examples	Benefit	Risks	Key refs
Antigen escape	Genetic	Dual targeting (e.g., CD19+CD22)	Covers heterogeneous/antigen-low clones; reduces antigen-negative relapse	More on-target/off-tumor risk; more tonic signaling/complexity	(19)
	Epigenetic – transcription factor	c-Jun overexpression program	Resists exhaustion under chronic stimulation; sustains pressure so escape variants don’t outgrow	Higher inflammatory output/tissue damage risk	(20)
	Metabolic	Inosine supplementation/refueling	Maintains effector function under glucose restriction	Tumor cells may also use inosine (could feed tumor)	(21)
	Micro-environment	“Armored” cytokines (IL-12/IL-18)	Recruits/activates bystander immunity; can clear antigen-low/loss lesions	Cytokine-mediated systemic toxicity	(22, 23)
Poor trafficking/infiltration	Genetic	Add chemokine receptor matching tumor chemokines (e.g., CCR2)	Improves homing to chemokine-rich tumors	Mis-trafficking to normal inflamed tissues expressing ligand	(24)
	Epigenetic -State	Manufacture toward memory-like states (less terminal differentiation)	Better persistence and migratory competence vs short-lived effectors	Slower initial tumor debulking; more variability by protocol	(2)
	Metabolic	Bias toward mitochondrial fitness (memory-like energetics)	Supports survival and repeated migration in hostile tumor microenvironment	May trade peak cytotoxic burst for durability	(25, 26)
	Microenvironment	Extracellular matrix barrier cutting (ie, heparanase expression)	Improves penetration in stroma-rich solid tumors	Extracellular matrix degradation can cause collateral tissue effects	(27)
Exhaustion/terminal fate	Genetic	c-Jun overexpression	Exhaustion resistance; improved function *in vivo*	More inflammation; potential autoimmunity/off-tissue injury	(20)
	Epigenetic/TF	NR4A knockout (exhaustion program repression)	Shifts chromatin/effector programs toward functional state	Removes a natural “brake” → damage risk in inflamed tissues	(28)
	Metabolic	Mitochondria-targeted rescue strategies	Can restore function in exhausted T cells	Could also prolong harmful activity if mis-targeted	(29)
	Microenvironment	PD1–CD28 “switch receptor”	Converts PD-L1 suppression into costimulation in TME	Breaks tolerance in PD-L1 tissues; inflammatory toxicity	(30)
Metabolic collapse	Genetic	ADA1/CD26 “localized refueling”	Converts adenosine→inosine; supports CAR T function in TME	Not automatically safe: inosine can be shared; tumor fueling risk not zero	(21, 31)
	Epigenetic	Epigenetic induction of mitochondrial biogenesis	Improves OXPHOS capacity and stress tolerance	Unknown translation to CAR context; may alter differentiation	(32)
	Metabolic	Inosine-driven support of stemness/fitness	Generates more persistent/potent CAR T phenotype	Substrate might benefit some cancers; context-dependent	(21, 33)
	Microenvironment	Target adenosine axis/shift purine balance	Reduces a dominant suppressive metabolite pathway	Adenosine is also anti-inflammatory “brake” in tissues	(33, 34)
Suppressive ligands/myeloid suppression	Genetic	PD1–CD28 switch	Overcomes PD-L1 mediated inhibition	Converts a safety checkpoint into activation → collateral damage	(30)
	Epigenetic/TF	c-Jun or NR4A editing	Resistance to dysfunction cues in suppressive TME	Harder to shut down once activated	(20, 28)
	Metabolic	ADA1 refueling against adenosine suppression	Directly counteracts adenosine-mediated suppression	May perturb nearby immune cells in tight-contact tissues	(31)
	Microenvironment	IL-12 TRUCK-like remodeling of myeloid/TME	Can flip suppressive myeloid circuits; broaden killing	IL-12 is potent—systemic spillover is dangerous	(22)
Systemic toxicity (CRS/ICANS)	Genetic	GM-CSF neutralization/KO in CAR T	Reduces CRS/neuroinflammation drivers	Might alter host defense; may change efficacy dynamics	(35)
	Epigenetic -Signaling	Costim domain choice/tuning (CD28 vs 4-1BB)	Can shift expansion/phenotype and cytokine profile	Tradeoff: speed vs persistence; toxicity profiles differ	(26, 36)
	Metabolic	Metabolic wiring differs by costim domain	Links CAR design → glycolysis vs mito fitness; impacts inflammation/persistence	Misaligned metabolism can worsen toxicity or failure	(26)
	Micro-environment -Clinical	CRS mitigation strategies (supportive + cytokine blockade)	Lowers mortality and enables dose intensity	Can blunt antitumor activity; timing-sensitive	(37)
Autoimmune tissue damage	Genetic	CAAR-T (autoreactive B-cell targeting)	Disease-specific deletion or durable remission in refractory autoimmunity	Infection/hypogammaglobulinemia; relapse mechanisms still possible	(9, 12)
	Epigenetic/Lineage	CAR-Treg approaches (stability/FOXP3 program)	Restores tolerance rather than blunt depletion	Treg instability could convert into pathogenic effector behavior	(15, 38, 39)
	Metabolic	Don’t blindly disable adenosine sensing	Adenosine restrains immunopathology in tissues	A2A pathway loss can worsen inflammatory tissue injury	(34)
	Microenvironment	Tissue contact effects (metabolite sharing/bystander shifts)	Explains why the same edit is safe in one tissue and risky in another	Tight cell–cell contact could amplify bystander modulation	(33)

## 2 Genetic reprogramming: from stronger signaling to smarter control of the CAR

CAR design is often framed as a search for “more activation,” but uncontrolled activation is a direct path to exhaustion and toxicity. The extracellular binder, hinge and transmembrane domains influence synapse geometry, antigen sensitivity, and tonic signaling; the intracellular domain dictates not only immediate killing but also differentiation trajectory (1, 2). Costimulatory modules encode biological bias: CD28-based CARs often drive rapid effector differentiation and cytokine production, whereas 4-1BB–based CARs tend to promote persistence and mitochondrial fitness, with consequences for both efficacy and inflammatory risk (40).

**Figure 1:**
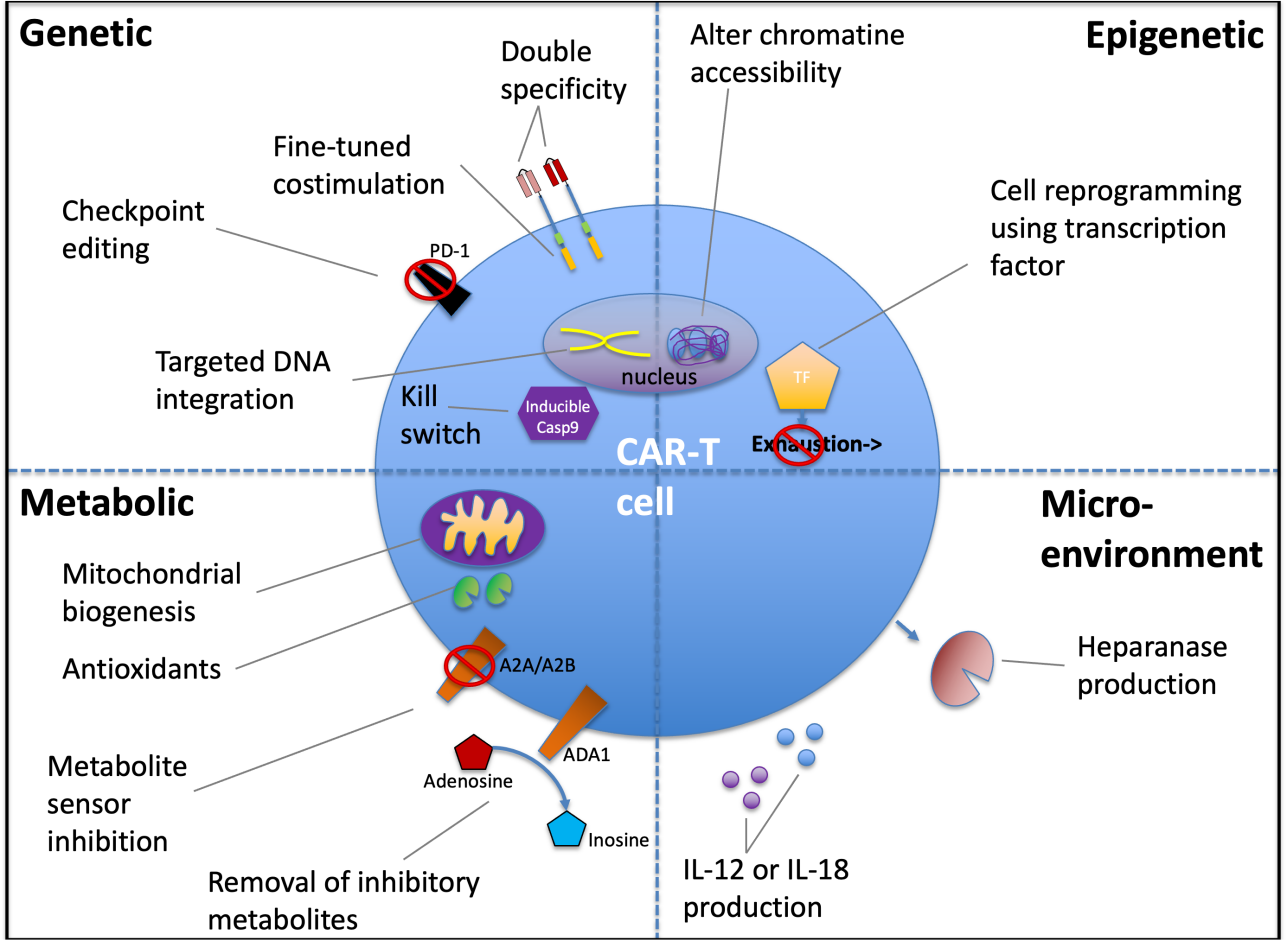
Multi-axis engineering of CAR-T cells to improve function in hostile tissues while preserving safety. Schematic overview of representative interventions organized into four layers (dashed quadrants) that can be combined to enhance CAR-T activity in solid tumors and other hostile tissues while maintaining controllability. Genetic strategies include multi-antigen targeting (double specificity), fine-tuned costimulation, checkpoint editing (illustrated by PD-1 disruption), and targeted DNA integration to standardize transgene expression. A built-in kill switch (e.g., inducible caspase-9) provides an emergency off-mechanism to mitigate severe toxicity. Epigenetic approaches aim to alter chromatin accessibility and reprogram cell state (e.g., transcription factor–based programming) to promote persistence and limit progression toward dysfunctional/exhausted phenotypes. Metabolic interventions enhance fitness through mitochondrial biogenesis and antioxidant support, counter purinergic immunosuppression via metabolite sensor inhibition (e.g., A2A/A2B adenosine receptor pathway), and reduce inhibitory metabolites through adenosine conversion to inosine (illustrated by ADA1). Microenvironmental modifications target tissue barriers and local suppression, including improved matrix penetration (heparanase production) and delivery of locally acting cytokine payloads (e.g., IL-12 or IL-18). Elements shown are illustrative rather than exhaustive and emphasize the principle that potency-enhancing edits should be paired with safety and control layers to manage tissue-specific risk. *Abbreviations:* CAR-T, chimeric antigen receptor T cell; TF, transcription factor; PD-1, programmed cell death protein-1; ADA1, adenosine deaminase-1; A2A/A2B, adenosine receptor A2A/A2B; iCasp9, inducible caspase-9; IL-12/IL-18, interleukin-12/interleukin-18.

Signal tuning is moving toward modular control rather than binary “on/off.” Next-generation CAR engineering emphasizes systematic optimization of hinge/transmembrane composition, immunoreceptor tyrosine-based activation motif (ITAM) number/position, and inclusion of alternative costimulatory domains (e.g., ICOS, OX40) to reduce tonic signaling and reshape cytokine profiles (41) (**Figure 1**). The standard for “improvement” should not be *in vitro* killing at saturating antigen; it should be a better efficacy–safety ratio under physiologic antigen density and tissue stress (42).

Targeted genome integration makes expression itself an engineering variable (**Figure 1**). Insertion of CAR constructs into defined loci such as the T-cell receptor alpha constant (TRAC) locus can standardize expression, reduce tonic signaling, and improve antitumor activity compared with random viral integration (43). For allogeneic “off-the-shelf” products, multiplex edits can disrupt endogenous T-cell receptor (TCR) to reduce graft-versus-host disease, while other edits can mitigate host rejection; however, each added edit increases manufacturing complexity and creates additional, poorly mapped interactions with host immunity. Recent base-editing strategies deserve mention here because they can reduce the double-strand-break burden during multiplex gene disruption, an attractive property when allogeneic products require several simultaneous edits. A recent example is a 2025 EGFR-targeting allogeneic CAR-T platform incorporating six engineered modifications spanning CD3E, B2M, CIITA, ADORA2A, PDCD1, and TGFBR2 to limit alloreactivity and resist biochemical and immunological suppression in solid tumors (44). Importantly, this study used a hybrid multiplex gene-editing strategy that combined adenine base editing with CRISPR-Cas12b nuclease engineering (44). The main lesson is therefore not that complex allogeneic manufacturing is now completely double-strand-break-free, but that base editing offers a way to lower genome disruption for multiplex knockout steps while enabling more ambitious resistance programs against the tumor microenvironment.

Logic-gated and titratable circuits are the clearest route to safer potency, especially for solid tumors and for autoimmune targets shared with essential immune compartments. AND-gated systems—dual CARs or synNotch→CAR cascades—require two antigens for full activation, reducing activity in healthy tissues that express only one antigen (45). Mechanistically, these systems are not all equivalent. In split-recognition dual CAR designs, one receptor delivers CD3ζ and the other costimulation, so full activation depends on co-encounter of both antigens (46). synNotch→CAR cascades add a transcriptional checkpoint: recognition of antigen A induces *de novo* expression of a CAR directed against antigen B, thereby confining cytotoxicity to tissues with the correct antigenic context (47). This logic can be used either to sharpen combinatorial specificity or to impose an antigen-density filter, as shown in synNotch circuits that discriminate HER2-high tumors from normal HER2-low tissues. Recent examples have also extended synNotch programs beyond CAR induction itself, including circuits that license local extracellular-matrix degradation only after tumor encounter, thereby improving infiltration while avoiding constitutive stromal remodeling (48). The key value of AND-gated systems is therefore not simply stronger function, but a wider therapeutic window; their trade-offs are activation delay, antigen-order dependence, and the creation of new escape routes if either antigen is heterogeneous or lost. NOT-gates (inhibitory CARs) convert recognition of a “protected” antigen into dominant inhibition (49). Small-molecule–gated split CARs and ON-switch platforms allow clinicians to titrate activity and potentially “pulse” therapy rather than commit to uncontrolled persistence (50). These tools address the core clinical constraint in both solid tumors and autoimmunity: the therapeutic window.

Safety switches should be standard equipment when pursuing high-risk targets or stacking potency edits (**Figure 1**). Inducible caspase-9 is a clinically validated “kill switch” capable of rapidly eliminating engineered cells if severe toxicity occurs (51). While this is not directly linked to CAR-T efficacy, these kind of design improvement will become mandatory with the diffusion of CAR therapies, in order to cure rare adverse event such as CAR-induced T lymphoma (52). Preventive safety can also be engineered by dampening inflammatory amplification. Mechanistic work implicates monocyte-derived cytokines, including IL-1 and IL-6, as key drivers of CRS/ICANS cascades (53); strategies that reduce myeloid activation (e.g., limiting GM-CSF signaling) can therefore reduce toxicity without necessarily compromising tumor killing, although the balance is context-dependent.

Checkpoint and cytokine resistance is where the field most often rationalizes risk. PD-1 or transforming growth factor-β (TGF-β) pathway disruption can enhance function in suppressive tumors (54, 55), but both pathways also restrain immunopathology. Their broad inhibition (i.e., PD-1 blockade with a monoclonal antibody) may increase tissue damage, accelerate terminal differentiation, or promote uncontrolled inflammation in autoimmunity (56). A safer design pattern is conditional resistance (e.g., switch receptors that re-route inhibitory signals into costimulation, or activation-dependent expression of dominant-negative receptors) combined with titratable activation and kill switches.

## 3 Epigenetic reprogramming: rewriting exhaustion and stabilizing desired fates

Exhaustion is characterized by a transcriptional and chromatin state induced by chronic stimulation, associated with reduced proliferative capacity, altered effector function and limited reprogrammability (57). This helps explain why checkpoint blockade can transiently improve function but often fails to durably reset exhausted programs: it modulates signaling without fully rewriting the underlying epigenetic landscape. For CAR T therapy, the engineering question is how to preserve a renewable pool of functional cells while enabling sufficient effector differentiation at the right time and place.

Direct transcription factor engineering can shift this balance. Overexpression of c-Jun counteracts exhaustion-associated AP-1 imbalance and enhances persistence and antitumor activity in preclinical models (20) (**Figure 1**). Deletion of exhaustion-associated transcription factors such as the NR4A family similarly improves CAR T function in solid tumors (28). Several other transcription factors and signaling pathways could also be considered to alter CAR-T cell features and exhaustion (CREM, CaMK4, Sirtuins, PPP2R2D) (58–62). These are exciting avenues for CAR therapies improvement, but they come with predictable risks. Exhaustion blockade may lead to unregulated activation, with increasing tonic signaling, cytokine secretion, or uncontrolled proliferation. In cancer, some of this risk may be tolerable with safety switches. In autoimmunity, the same edits could amplify tissue damage or convert a tolerogenic intent into inflammatory disease.

Manufacturing-time programming is often dismissed as “process,” yet it is one of the most reliable epigenetic levers available. *Ex vivo* culture conditions imprint long-lived chromatin accessibility and differentiation trajectories. PI3K/AKT pathway inhibition during expansion can preserve less-differentiated memory-like phenotypes and improve antitumor activity after transfer (63). The mTOR pathway is also a druggable pathway which leads to deep modification in T cell function with improvement of their fitness, proliferation and function (64). Clinically, this implies that “next-generation” function can sometimes be achieved without additional genome edits, which matters for manufacturability and safety. For autoimmune applications, manufacturing choices may be even more consequential: maintaining naïve/memory features could support durable immune reset at lower doses (65), while for CAR-Tregs the goal is stable suppressive identity and avoidance of conversion to effector phenotypes.

Epigenetic reprogramming is also relevant through inadvertent experience in patients. Clinical observations have linked disruption of epigenetic regulators such as TET2 to altered CAR T expansion and persistence, underscoring how chromatin modifiers can rewire fate (66). The lesson is not that TET2 should be routinely disrupted, but that epigenetic state is a causal variable that can dominate outcome. Future strategies may shift toward targeted epigenome editing (CRISPR–dCas9 fused to chromatin modifiers) to silence exhaustion-associated enhancers or reinforce tolerance loci without introducing DNA breaks. This is conceptually attractive for autoimmunity, but delivery and off-target epigenetic effects remain major gaps that will need to be addressed before implementation.

## 4 Metabolic reprogramming: make cells compatible with the tissue they must survive in

Metabolic stress is a major contributor of CAR T failure in solid tumors and may shape altered function in inflamed autoimmune tissues (67). Tumors impose hypoxia, reactive oxygen species, acidity and nutrient scarcity while accumulating immunosuppressive metabolites such as adenosine and lactate; these conditions directly impair T-cell proliferation and effector function (68–70). A central problem is that CAR T cells are typically selected and expanded under nutrient-rich, oxygenated conditions that train them for the wrong environment. Metabolic programming therefore must be assessed under physiologic stress, or it will not translate.

Costimulatory biology and metabolism are inseparable. CD28 versus 4-1BB signaling drives distinct metabolic programs that influence persistence, cytokine output and exhaustion sensitivity (68). Beyond this, mitochondrial fitness has emerged as a key lever because it integrates energy production, redox signaling and apoptosis sensitivity. A recent review emphasizes that mitochondrial dynamics (fusion–fission balance) regulate effector versus memory-like phenotypes and influence migration; it also highlights intercellular mitochondrial transfer within the tumor microenvironment as an underappreciated mechanism that can either drain T-cell bioenergetics or, if therapeutically directed, restore function (71). This matters because it reframes “tumor microenvironment suppression” as partly a bioenergetic tug-of-war, not just cytokine signaling.

Selective refueling provides a concrete example of metabolic engineering aimed at both efficacy and immunosuppression (**Figure 1**). Extracellular adenosine should be viewed not merely as a generic suppressive metabolite, but as part of a physiological tissue-protective pathway that limits excessive inflammation (72, 73). Foundational studies first established adenosine receptor signaling as an endogenous brake on inflammatory tissue damage and then showed that hypoxic tumors co-opt the A2A receptor pathway to suppress incoming antitumor T cells. In the CAR-T setting, this axis later became a direct engineering target when CRISPR/Cas9-mediated A2AR deletion was shown to enhance CAR-T effector function and *in vivo* efficacy (74).

Hu and colleagues engineered CAR T cells to overexpress CD26 and cytoplasmic adenosine deaminase 1 (ADA1), converting immunosuppressive adenosine into inosine, an alternative carbon source when glucose is scarce (31). Fusion of ADA1 to an anti-CD3 single-chain variable fragment aimed to localize enzyme activity to T cells and reduce tumor fueling; the engineered cells showed improved migration and resistance to TGF-β–mediated suppression in mouse solid-tumor models (31). The strategic insight is that metabolic interventions can double as microenvironmental interventions when they simultaneously remove suppressive metabolites (adenosine) and provide usable substrates (inosine). Several other key metabolic enzymes have been shown to deeply affect T cell function and could be considered (PFKP, PP2A, Glutaminase 1/2…) (75–78). Alternatively, providing CAR-T cell with detoxifying equipment to fight ROS may reinforce their stability (79–81).

Metabolic engineering is not automatically safe. Inosine is not a private resource; some tumor cells can use it (21). Localizing ADA1 to T cells reduces but may not eliminate the risk of feeding tumors or altering bystander immune cells, particularly in tissues with tight cell–cell contact (31). More broadly, disabling adenosine sensing (e.g., A2A receptor knockout) may increase inflammatory damage because adenosine also restrains excessive immune activation (34). Thus, metabolic edits should be paired with safety switches or titratable circuits, and they should be evaluated for tissue-specific risk in autoimmunity. Interestingly, these extrinsic metabolic improvements may be used to improved T cell functions also in the setting of T-cell engager therapy (82).

## 5 Microenvironmental reprogramming: combine intrinsic engineering with niche remodeling

Even well-programmed cells fail if the battlefield blocks access. Solid tumors present physical exclusion (dense extracellular matrix, abnormal vasculature), suppressive cytokines (TGF-β, IL-10), checkpoint ligands (PD-L1), and suppressive myeloid networks that deplete nutrients and generate reactive oxygen species (71). Approaches that ignore these barriers tend to compensate with higher doses and stronger signaling—exactly the recipe for systemic toxicity.

Trafficking and penetration can be engineered directly. Enzymatic matrix traversal (e.g., heparanase expression) can improve infiltration in solid tumor models (27), but introduces off-tumor risks because extracellular matrix components and stromal targets are not tumor-exclusive (**Figure 1**). Matching chemokine receptor expression to tumor chemokine gradients is a complementary strategy that can reduce required cell numbers; CCR2 expression to follow CCL2-rich tumors is a canonical example (24). These approaches should be evaluated not only for increased tumor infiltration, but for whether they shift biodistribution into sensitive healthy tissues.

Localized immunomodulation can reshape hostile niches without systemic toxicity if it is truly localized. “Armored” CAR T cells secreting cytokines such as IL-12 or IL-18 can recruit endogenous immunity and convert suppressive microenvironments into inflammatory ones (22, 23). The safety pitfall is leakage: constitutive cytokine secretion can reproduce CRS-like syndromes. The rational direction is inducible, synapse-restricted, or dual-antigen–gated payload release rather than constitutive secretion, coupled with kill switches as backstops. Similarly, external combinations (radiotherapy, oncolytic viruses, myeloid-targeting drugs) should be selected not by tradition but by mechanistic fit. The mitochondrial metabolism literature underscores that some combinations may fail by imposing oxidative stress that harms CAR T viability, implying that “niche remodeling” must include protection of CAR T bioenergetics, not just tumor sensitization (71).

One under-discussed strategy is to reprogram the microenvironment before adoptive transfer rather than forcing the engineered cell to solve every barrier intrinsically (83). Work on the hypoxia-adenosinergic axis has shown that supplemental oxygenation and antihypoxic strategies can weaken a major biochemical checkpoint that suppresses T-cell effector function in solid tumors (84). More recently, oxygen-carrying perfluorocarbon nanoemulsions combined with respiratory hyperoxia were reported to eliminate tumor hypoxia, increase intratumoral infiltration of activated T cells and NK cells, and improve the efficacy of endogenous or adoptively transferred T cells (85). Because these data are not yet uniformly CAR-T specific, they are best presented as a rational preconditioning strategy for adoptive cell therapy/CAR-T rather than established CAR-T practice. Conceptually, however, they are highly relevant to a failure-mode framework: upstream remodeling of tumor biochemistry may reduce the amount of intrinsic overengineering required in the cell product itself.

Autoimmunity raises a different microenvironment problem: chronically inflamed tissues and lymphoid architecture may sustain pathogenic memory (86). Engineering goals shift from “infiltrate a tumor mass” to “find and reset the pathogenic niche” (germinal centers, tertiary lymphoid structures, target organs) while preserving host defense (10). This favors tissue-homing programming, regional delivery, titratable activity, and time-limited persistence. It also argues against importing cancer-style constitutive pro-inflammatory armoring into autoimmune settings. For CAR-Tregs, microenvironmental programming is partly about survival and stability in inflamed tissues (38); engineering resistance to inflammatory cytokines must be balanced against the risk of uncontrolled suppression.

## 6 Discussion and outlook

A proportion of CAR therapies failures stem from mismatches between the product and the environment. The field’s most expensive mistake is treating efficacy and safety as separate optimization problems. Potency edits that remove brakes, amplify cytokines, or extend persistence can quickly shift toxicity from manageable to catastrophic, in both cancer and autoimmunity fields.

In autoimmunity, the endpoint is not maximal cytotoxicity but deep depletion of autoreactive B cell subsets with minimal irreversible immunodeficiency, which means that the antigen targeted (CD19 vs BCMA), tissue distribution, persistence, and target-compartment choice matter at least as much as raw potency.

A failure-mode–driven approach is more disciplined than technology-driven iteration. Antigen escape points to multi-antigen recognition and retargetable platforms. Trafficking failure points to chemokine matching, matrix traversal, and regional delivery. Metabolic collapse demands testing under physiologic stress and considering interventions such as inosine refueling or mitochondrial fitness control. Toxicity constraints demand titratable circuits and validated safety switches as standard components, not optional accessories.

The next generation of CAR therapies will likely be closed-loop systems that sense context (hypoxia, adenosine, inflammatory cytokines) and compute responses—kill, suppress, expand, or shut down. Progress will be measured not by the number of edits, but by whether multi-layer reprogramming can deliver durable benefit with predictable, controllable risk in both cancer and autoimmune disease.
